# Self and Dyadic Management of Patients and Care Partners Living with Durable Ventricular Assist Device: Emerging Science, Studies, and Frameworks to Advance Precision Health

**DOI:** 10.1007/s11897-025-00737-6

**Published:** 2025-12-20

**Authors:** Kristin E. Sandau, Stacy A. Al-Saleh, Christin Quarry, Samantha Conley

**Affiliations:** 1 University of Minnesota (UMN), Adult and Gerontological Health Cooperative, School of Nursing, 5-140 Weaver-Densford Hall, 308 Harvard St SE, Minneapolis, MN 55455 USA; 2 Division of Nursing Research, Mayo Clinic, Phoenix, AZ USA; 3 Division of Nursing Research, Mayo Clinic, Rochester, MN USA

**Keywords:** Dyads, Dyadic LVAD management, Self-management, Heart assist device, Left ventricular assist device, LVAD, Caregivers, Care partners, Self and family management

## Abstract

**Purpose:**

The goal of this paper is to advance precision health among patients with a left ventricular assist device (LVAD) and their informal care partners by examining the science of patient, care partner and dyadic management. We (1) describe the current state of self- and dyadic management science in LVAD, and (2) offer frameworks to guide LVAD patient, care partner and dyadic management research.

**Recent Findings:**

While frameworks for family and self-management exist for other chronic conditions, dyadic frameworks are rarely used for LVAD patient-care partner dyads, thus limiting research. A handful of primarily qualitative studies describe self-management tasks and skills, but facilitators, barriers, processes, behaviors and outcomes of self- and dyadic LVAD management need more study.

**Summary:**

In this paper we summarized current progress in self- and dyadic management science in LVAD and offered a preliminary framework to guide research. Future studies should include both care partners and LVAD patients, with thoughtfully selected frameworks to guide more inclusive research in LVAD self- and dyadic management, with the goal of designing the *right intervention* for the *right person* at the *right time*.

**Supplementary Information:**

The online version contains supplementary material available at 10.1007/s11897-025-00737-6.

## Introduction

Heart failure is a chronic condition that affects 55.5 million people worldwide [1]. In the United States, approximately one in four individuals will develop heart failure in their lifetime [2]. For individuals with advanced heart failure, limited donor hearts are available, precipitating the use of a surgically implanted left ventricular assist device (LVAD) [3]. An LVAD may be indicated as a bridge to transplant, as a bridge to recover from a catastrophic cardiac event, while awaiting decision about transplant listing, or as long-term heart failure management (“destination therapy”) [3]. Over a ten-year period starting in 2014, well over 27,000 LVADs were implanted worldwide [4], requiring these individuals to learn LVAD self-management (also referred to as self-care in the literature).

*Self-management* encompasses the day-to-day knowledge, skills and behaviors that people undertake to manage their health [5]. LVAD self-management includes assessing and changing driveline dressings using aseptic technique, keeping the controller dry during showering, managing devices, alarms, medications, dietary changes, medical appointments, as well as self-management adjustments in broader areas of life such as emotional and social, which are less often studied [6–8]. However, people living with an LVAD do not typically manage their LVAD alone; thus, thousands of non-clinicians provide support for individuals living with an LVAD, with this caregiver-patient relationship typically resulting in the formation of an LVAD management dyad. In this paper, we use the term *dyadic LVAD management,* defined as a collaborative process where two people, comprising patient–care partner dyads, manage a chronic condition together and the degree to which they share LVAD management.

LVAD caregivers (hereafter referred to as care partners) are essential for preventing death and life-threatening complications (e.g., infection, bleeding, and stroke) post-LVAD implant [9, 10]. Care partners for LVAD patients are informal supporters (typically unpaid spouses, family, or friends) who perform LVAD self-management with the LVAD patient. The critical nature of the care partner role is emphasized by the International Society for Heart and Lung Transplantation (ISHLT) guidelines, which state that the lack of a care partner is a relative contraindication to LVAD implantation [11]. The presence of a highly trained care partner reduces mortality of LVAD patients; notably, the risk of patient death tripled for those who lived alone [9]. In a U.S. study, LVAD patients whose care partners quit this role experienced higher thirty-day readmission rates [10].

Dyadic LVAD management also impacts care partner outcomes. While the LVAD care partner improves the outcomes of the LVAD patient, this role can come at a high individual cost to the care partner. Care partners report feeling unprepared and overwhelmed [12], symptoms of depression and anxiety [8], reduced quality of life [13], and significant caregiver burden [14], which do not typically resolve with time [8, 15]. It is essential to recognize that the LVAD care partner and patient are both integral for LVAD success and optimize outcomes for the LVAD patient, care partner, and dyad [13, 14].

The importance of care partner support and dyadic management in heart failure [16] is well-documented. Riegel et al. [17] explicated self-care as including the processes of maintenance, symptom perception, and management as being integral to self-care among people with heart failure, with social support conceptualized as an environmental factor that influences the process of self-care. [18]. Grey and colleagues [19] stressed that self-management rarely occurs at the individual patient level but instead occurs within the context of the family, elaborating the science of Self- and Family Management in Chronic Illness [20]. The usefulness of considering dyadic aspects of self-management was confirmed by the work of Casida and colleagues [21] highlighting the need to better understand that LVAD management occurs beyond the single “patient.”. To advance the science of LVAD dyadic management it is essential to consider patients, care partners, and their dynamic interaction with a conceptually grounded approach. A more precise understanding of LVAD self-management is needed for those along their perioperative LVAD trajectory as both the patient and care partner “live” with the LVAD. Precision health encompasses health strategies that allow a patient, with a care partner(s), in collaboration with their health care clinician, to select interventions to align with a person’s and a dyad's unique circumstances by accounting for social, cultural, economic, and emotional factors that can influence their health and quality of life [22]. Due to the high burden of LVAD dyadic self-management it is essential to identify the *right self-management intervention* for the *right person* at the *right time*.

## Purpose of Paper

The goal of this paper is to advance precision health among LVAD patients and their informal care partners by examining the science of dyadic LVAD management. We (1) describe the current state of dyadic LVAD management science in LVAD, and (2) offer frameworks to guide dyadic LVAD management research and practical interventions for LVAD care partners and patients.

## Literature Review

To inform the development of a conceptual framework to guide a study about dyadic LVAD management, we conducted a purposeful review of studies that described self-management or self-care among care partners and LVAD patients to understand the current state of dyadic LVAD management science. We searched LVAD AND self-care OR self-management AND caregiver OR dyad using a combination of keyword and subject heading approaches (see Supplementary Table 1 for search strategies). Medline, Embase, Cochrane Central Register of Controlled Trials, and Cochrane Database of Systematic Reviews databases were searched via Ovid. Searches were limited to English language, adults, with publication date from 2015 to March 2025. Studies were included when self-management or self-care (as these terms are often used interchangeably) was reported for both LVAD patients and LVAD care partner or the dyad. We excluded reports that were abstract-only, and non-research clinical papers, or opinion papers.

We included 6 studies, which consisted of 7 reports in our review (Table 1). Sample sizes ranged from 12 to 218. Most patients were male; most care partners were female. Studies included LVAD patients that received their devices for both long-term heart failure management and as a bridge to transplant. The included studies were primarily descriptive, and of these, four were qualitative,[23, 24, 28, 29] and one was a randomized clinical trial [25].

**Table 1:** Summary of study characteristics for LVAD patient and care partner self-management

Authors (year), reference Country	Study Design	Sample Size	Care partner relationship to patient	Female N (%)	Race/Ethnicity	Age (years) Mean (SD)	LVAD Characteristics
Abshire et al. [23] USA	Qualitative (interviews and photographs) in home settings *(Matched dyads) Interviewed separately, unless requested to interview together*	10 LVAD patients 10 Care partners	NR	**Patient** 4 (40%) **Care partner** 8 (80%)	**Patient** African American 4 (40%) White 5 (50%) Asian 1 (10%) **Care partner** African American 4 (40%) White 5 (50%) Asian 1 (10%)	**Patient** 55.8 (12.8) **Care partner** 52.6 (14.0)	Destination therapy: 7 (70%) Bridge to Transplant: 3 (30%) Time from implant: < 1 year: 8 (80%)
Barsuk et al. [24] USA	Qualitative *(Nondyadic sample; not paired)*	16 LVAD patients 12 Care partners	NR	**Patient** 6 (37.5%) **Care partner** 10 (83.3%)	**Patient** White 9 (56.3%) African American 6 (37.5%) Other 1 (6.3%) Hispanic or Latino/a 1 (6.3%) **Care partner:** White 6 (60%) African American 5 (50%) Other 1 (10%) Hispanic or Latino/a 1 (10%)	**Patient** 53.1 (14.2) **Care partner** 51.3 (14.3)	Destination therapy: 9 (56.3%) Bridge to transplant: 7 (44.5%) (At time of interview) Time from implant: 31% were < 6 months; 31% were 6–24 months; 38% were > 24 months)
Barsuk et al. [25] USA	Randomized pilot trial *(Nondyadic sample; not paired)* Simulation-based mastery learning curriculum (SBML)	40 LVAD Patients 40 Care partners	Spouse/partner 22 (55.0%) Child 6 (15.0%) Parent 3 (7.5%) Sibling 8 (20.0%) Other 1 (2.5%)	**Patient** 11 (27.5%) **Care partner** 31 (77.5%)	**Patient** Black 12 (30.0%) American Indian/Alaskan native 1 (2.5%) Asian 2 (5.0%) White 25 (62.5%) Hispanic/Latino 2 (5.0%) **Care partner** Black 13 (32.5%) American Indian/Alaskan native 1 (2.5%) Asian 2 (5.0%) White 24 (60.0%) Hispanic/Latino 1 (2.5%)	**Patient** 55.2 (12.9) **Care partners** 52.35 (14.6)	Destination therapy: 28 (70.0%) Bridge to transplant: 12 (30.0%)
Casida, Combs, Pavol et al. [26] Casida, Combs, Schroeder et al. [27] USA	Mixed Methods Study *(Nondyadic sample)*	102 LVAD patients 116 Care partners	NA	**Patient** 35 (34%) **Care partner** 94 (81%)	**Patient** Asian: 2 (2%) Black 17 (17%) Hispanic Non-White: 1 (1%) White 80 (78%) Mixed 1 (1%) Native American 1 (1%) **Care Partner** Asian: 4 (3%) Black 14 (13%) Hispanic Non-White: 2 (2%) White 94 (80%) Mixed: 1 (1%) Native American: 0	**Patient** 51.4 (13.8) **Care partner** 48.9 (12.7)	Bridge to Transplant: 70% Destination therapy: 20% Bridge to Recovery: 10% Time from implant: 2–74 months (mean 19.7 ± 15 months)
DeGroot et al. [28] USA	Qualitative *(Matched dyads)*	10 LVAD Patients 10 Care partners	NR	**Patient** 4 (40%) **Care partner** 8 (80%)	**Patient** White 5 (50%) African American 4 (40%) Asian: 1 (10%) **Care Partner** White 5 (50%) African American 4 (40%) Asian: 1 (10%)	**Patient** 55.8 **Care partner** 52.6	Indication: NR Time from implant: 3–12 months (or, if greater, hospitalized within past 30 days)
Rapelli et al. [29] Italy	Qualitative *(Matched dyads) who were in cardiac rehabilitation*	6 LVAD Patients 6 Care partners	Spouse: 3 (50.0%) Cousin 1 (16.7%) Brother 1 (16.7%) Mother 1 (16.7%)	**Patient** 0 (0%) **Care partner** NR	NR	**Patient** 48.79 **Care partner** 51.1	Indication: NR

*Note:* NA = not applicable, NR = not reported

## LVAD Patient Management

LVAD patient self-management refers to how living with an LVAD includes extra responsibilities beyond an original heart failure diagnosis (i.e., perioperative time off work or other role changes, aseptic driveline dressing changes, modified showering, alarm management). In the studies reviewed (Table 2), LVAD patients did not manage their LVAD alone. LVAD patients reported that they continued to share LVAD management with their care partners even when they had high LVAD skills, knowledge and confidence, and wanted increased independence [23, 24, 29]. LVAD patients desired refresher training of LVAD skills from LVAD providers [26, 27]. Some patients felt like a burden on their care partner [23, 29]. Emotional, psychological, and spiritual self-management received minimal report, as did how patients managed LVAD complications.

**Table 2:** Focus of LVAD patient, care partner, and dyadic LVAD management in studies reviewed

Self-Management Focus	LVAD Patient	LVAD Care Partner	LVAD Dyad
Education needs	Desired hands-on demonstration [24] Wanted follow-up from provider [27].	Desired movies as resources to reference [24]. Wanted follow-up from provider [27]. Wanted refresher/retraining [24, 27].	Both suggested emergency and reminder cards (i.e., “how to”) [24]. Both felt education immediately prior to hospital discharge felt rushed [24]. Less than 5% of patients and care partners reported difficulty with retention of LVAD skills and knowledge, but the majority wanted refresher education within the first 6 months [27].
Medications and appointments	Uses reminders [23].	Organizes medication [24].	Care partner support was required; collaboration on managing medications and appointments using cellphone calendars, both setting smart phones. Cognitive aids varied from home monitoring to reminders on “sticky notes” [23].
Device management	Patient relied on care partner to perform driveline site care [24]. Needed help to manage the device [24, 29].; wanted more independence [23]. 90% were tested for LVAD skills; 62% were tested for LVAD knowledge. 75% felt prepared for discharge [26] Simulation training improved skills [25].	Care partner saw self as central for LVAD management [29]. Did dressing change until the patient was comfortable doing it and they assessed patient skills [23]. 97% were tested for LVAD skills; 56% were tested for LVAD knowledge. 75% felt prepared for discharge [26] Simulation training improved skills [25]. Experienced fear that alarm would go off [28].	Patients increased their independence but care partners remained involved [23].
LVAD complications	--	--	--
Partnership with providers and clinicians	--	--	Both reported that home care nurses lacked knowledge and skills for helping manage LVAD device [27].
Problem-Solving, Taking Action	Patients kept needed items by bed so within reach while LVAD plugged into electrical outlet at night; some used recliner instead of bed [23].	Many care partners changed sleeping arrangements (i.e., separate beds) [23].	Both worked to rearrange home to adapt for life with an LVAD [23] Most home adaptations occurred in bathrooms (removable shower heads, shower chairs, hanging hooks to hold LVAD equipment) [23]. Shared self-management tasks changed over time; some households lacked space and hygiene for optimal management [23].
Showering/bathing	Patients often were not satisfied with not being able to shower independently [23].	--	Care partner typically managed LVAD while patient cleaned themselves [23].
Confidence	Many patients (75%) felt ready for discharge [26]. Patient confidence with LVAD management increased with time [28]. Patients reported high self-confidence for performing LVAD-related tasks [25] Patients were concerned about how to manage the LVAD at home [29].	Most care partners (94%) felt ready for discharge [26]. Care partners were worried that they would not know what to do [28]. Care partners reported high self-confidence for performing LVAD-related tasks [25].	While patients gained increased confidence, care partners continued to have constant worry [28].
Role: Independence; independent activities of daily living (IADLs); family responsibilities Role: Self-identity; Employment	Many felt like burden on care partner [29]. Most wanted more independence (i.e., showering) [23] --	Many denied feeling that the patient was a burden to them [29]. Spousal/partnered care partners were highly involved in independent activities of daily living (IADLs), with non-spousal partners having more variation in approach to IADLs [23]. --	Care partner burden was reduced over time as patients became more independent, but care partners continued to be relied upon by many patients [23]. Spousal/partnered care partners were highly involved in IADLs; if the wife had been responsible for cooking and cleaning pre-implant, these responsibilities were continued post-implant [23]. --
Emotional, psychological, spiritual wellbeing	Concerns about what to expect/uncertainty (i.e., illness, waiting for transplant) [29].	Worry and uncertainty [28]. Drained from care; concerned about their ability to cope [29].	*Within dyads:* The most congruent perceptions included worry about home device management (driveline dressing changes, power supply, complications); the importance of the care partner role, which was characterized by stress, depression, role strain; loss of control; and uncertainty and about changes in role, relationship [29]. The least congruent perceptions included whether the patient was perceived as a burden (patients verbalized this; care partners denied it) [29]. Both described their spirituality as a meaningful coping strategy [29]. Both described being healthy and having a healthy family as good quality of life [23]. Both reported impaired QoL and effort to regain a sense of normalcy despite challenges [28].
Health promotion behaviors (i.e., nutrition, physical activity, sleep)	--	--	--
Goal-setting and decision-making	--	--	Only about half the dyads had congruence about thoughts/expectations for end of life [28].

*Note:* -- indicates that data are minimal or not available from studies reviewed

## LVAD Care Partner Management

LVAD care partner self-management refers to how care partners manage their own health and wellbeing in the context of additional responsibilities for their partner after LVAD implant. LVAD care partners saw themselves as central for LVAD management. After training in knowledge and skills, care partners felt more prepared for hospital discharge compared to LVAD patients. [26]. Many care partners reported ongoing concerns about LVAD management, including fear of device alarms [28]. Care partners also provided instrumental support such as rearranging the house, cleaning, cooking, and although patients set up medications, care partners provided reminders for pills and appointments [23]. Like LVAD patients, care partners reported a need and desire for retraining and ongoing support from LVAD providers, and did not find home care nurses supportive of LVAD self-management as they lacked LVAD skills [27]. How LVAD care partners managed appointments, LVAD complications, and their own physical and psychological health needs received minimal report.

## LVAD Dyadic Management

Building on the concept of dyadic management, many patients relied on care partners to perform driveline site care [23, 25]; however, in some dyads, patients became independent in dressing changes [23]. Spousal/partnered care partners were highly involved in independent activities of daily living (IADLs), with non-spousal partners and dyads with more complex needs having more variation in approach to IADLs management [23]. While confidence and independence of LVAD patients increased over time, most care partners continued to worry about how to support LVAD self-management [28]. Role, emotional, and spiritual aspects of dyadic self-management received little study, as did how medical and LVAD complications were managed.

In some areas of dyadic self-management, patients and care partners had shared worries and coping strategies (worry about device management; loss of control; uncertainty and changes in role and relationship; and spirituality as a meaningful coping strategy) [29]. However, dyads were less likely to share similar thoughts and expectations for end-of-life care planning [28]. In one study, several patients verbalized feeling like an imposition, although most care partners denied feeling this [29].

Most studies did not report dyadic findings, even when dyads were recruited, limiting conclusions about LVAD dyadic management. Additionally, while interventions for LVAD education included both the LVAD patient and care partner [25], it is unclear how the dyad navigated shared LVAD management or relationship and role changes. Data were lacking or absent about dyadic management for LVAD complications and common health behaviors (nutrition, physical activity, sleep).

## Gaps in the Literature

The literature about LVAD dyadic management is limited. Many processes are not yet understood (e.g., who is doing what management tasks and at what time points postoperatively; shared decision-making and adaptations made in routines). Dyadic management for medications and appointments was described, but was lacking for other health behaviors (i.e., nutrition, physical activity, sleep). Most included studies primarily focused solely on LVAD device self-management. Researchers have previously demonstrated that after LVAD implantation, measures for quality of life, anxiety, and depression for LVAD patients improve while their care partners outcomes get worse or stay the same [8, 15]. However, it is not known if this divergence of outcomes is due to the burden of dyadic LVAD management, watching a loved one experience advanced heart failure, post-traumatic stress disorder from the grueling surgery [30], experiences in the intensive care unit, feeling overwhelmed by the responsibility of supporting their partner’s life [12], or other life demands. To advance precision care and outcomes for patients, care partners, and the dyad following LVAD implantation, it is essential to use a conceptual framework that addresses the unique dyadic nature of LVAD management and the dynamic trajectory of experience with LVAD (pre- implant, perioperative, and varying stages post-implantation).

## Frameworks to Guide Dyadic LVAD Management Research

### Foundational Self-Management Theories

The understanding of self-management has evolved, based on Bandura’s work in *self-efficacy* [31] and Corbin and Strauss’s [32] description of the work of chronic illness as *three sets of tasks*, which Lorig and Holman [5] further explicated:

- Medical or behavioral management (e.g., taking medications, dietary choices);
- Role management (e.g., employment status, family responsibilities, self-identity, adapting hobbies to compensate for new disabilities); and
- Emotional management (e.g., dealing with emotional sequelae of chronic illness).

Lorig defined, operationalized, and tested self-management [5, 32–35]

further adding *five self-management skills*:

- Problem solving
- Decision making
- Finding/utilizing resources
- Partnerships with providers, and
- Taking action.

Complementing this work, Reigel and colleagues [17, 36] identified the processes of maintenance, symptom perception, and management as being integral to self-care among people with heart failure [18]. This framework has been applied in the context of LVAD, including an LVAD Care Behaviour Scale, but at an individual level [37, 38].

Recently Lyons and Lee [39] and Buck and colleagues [40] introduced conceptual work among heart failure dyads. In the Theory of Dyadic Illness Management [39] by Lyons and Lee, the adult patient-care partner dyad is the focus with the central goal of optimizing dyadic health. In this model, the dyad is an interdependent team, and the way the dyad appraises illness (either as congruent or incongruent) influences the ways in which they share management. Further, Buck and colleagues [40] described dyadic congruence by conceptualizing and operationalizing four different dyadic heart failure self-care typologies:

- Patient does all self-care
- Caregiver does all self-care
- Patient and caregiver collaborate
- Patient and caregiver complement each other.

Assessing the degree of dyadic congruence has potential to help clinicians avoid incorrect assumptions about who is typically responsible for what task or role. Further, recognition of dyadic typology can help mitigate goal nonattainment related to mismatch of goals. Researchers [39–41] have introduced dyadic approaches in heart failure, yet application in the unique context of LVAD patient-care partner dyads is unclear.

### Conceptual Framework for LVAD Dyadic Management

**Fig. 1:**
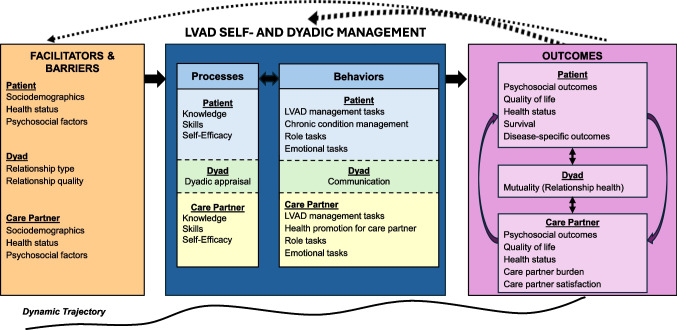
Conceptual framework for self- and dyadic management for LVAD patient-care partner dyads*.* Note: Adapted from Corbin & Strauss [32]; Lorig & Holman [5]; Grey et al. [19]; Schulman-Green et al. [20] and Lyons and Lee [39]. (Used with permission from Conley & Sandau.)

To guide an ongoing feasibility study characterizing LVAD dyadic management trajectories, we developed a conceptual framework that integrated and adapted foundational self-management conceptual work in chronic conditions [5, 20, 31, 32], dyadic management in heart failure [39, 40] and the LVAD empirical literature reviewed above. In this conceptual framework, we view *dyadic LVAD management* as a collaborative process where two people, comprising patient–care partner dyads, manage a chronic condition together and the degree to which they share appraisal, management knowledge, skills, and behaviors along a dynamic trajectory while living with an LVAD, with three important sets of outcomes: *patient, care partner, and dyad*. While recognizing the importance of dyadic outcomes, we remain concerned about individual care partner outcomes [12] with more than a decade of evidence for poor care partner outcomes; thus the framework displays specific outcomes at the individual as well as dyadic level. The *Conceptual Framework for Self- and Dyadic Management for LVAD Patient-Care Partner Dyads* has three main components: (1) Facilitators and Barriers, (2) LVAD Self- and Dyadic Management, and (3) Outcomes (see Fig. 1).

Along a dynamic trajectory pre-implant through post-implant, the first component, *Facilitators and Barriers*, includes individual characteristics of the care partner and the patient, as well as their relationship. The *type* of relationship (e.g., spouse, mother, adult son) will affect dyadic management. For example, a married dyad who lives together with school-aged children at home typically communicates and divides management tasks differently than an adult sister and brother dyad who live separately. Further, the *quality* of relationship at baseline is important as significant transitions and changes occur through lifespan and LVAD trajectory [42]. We cannot assume, however, that the married dyad will, by default, by demonstrate more congruent, collaborative patterns over time. Healthy relationship quality at baseline is likely to be a mediator for strong relationship quality sustained over the dynamic perioperative trajectory, but this cannot be assumed without longitudinal assessment. Mutuality may be both a protective factor that mitigates care partner decline as well as an outcome. Similar to self-efficacy, mutuality likely contributes both as precursor and outcome through feedback loops. Finally, clinicians recognize that the variability of patients in their own individual self-management pre-LVAD implant.

The second major component, *LVAD Self and Dyadic Management,* encompasses two parts: *Processes* and *Behaviors*. *Management Processes* include knowledge, skills and self-efficacy which are uniquely critical to living with an LVAD to ensure readiness for home LVAD self-management [11]. Intense learning is needed to master the device-related knowledge and skills in a short timeframe, often by the care partner while the patient is recovering in the intensive care unit (ICU) [12]. LVAD self-efficacy, which precedes self-management behaviors [31], has been reported for patients and care partners in regard to LVAD management [43]. In contrast, measures of LVAD care partner’s self-efficacy for the care partnering role have received minimal study, representing a missed opportunity for interventional studies targeting stress, coping, the transition to an unpaid care partner, and how care partners manage time to care for their own health. Emerging science underscores the importance of understanding dyadic appraisal [39] (i.e., how each perceives their ability to navigate a chronic condition or care partnering) and dyadic congruence [40] (i.e., how aligned are patient and care partner on their perceptions of appraisal; how satisfied is each with this arrangement). The degree to which self-management is “shared” varies tremendously among dyads – from the care partner doing very little to the care partner continuing to assist with diet, childcare, follow-up, driveline site care months or years after implantation. Thus, the dyadic appraisal – not just individual appraisal - is key.

In addition to *Processes*, the second part of *Management* includes *Behaviors*. We selected behaviors because they are measurable, influence health outcomes, and are required to sustain life. Patient LVAD self-management behaviors have been described in the literature [37]. However, less is known about health promotion for the care partner. Further, how dyadic communication and management impacts outcomes remains unexplored. Self-management tasks (role, and emotional) are navigated differently by the patient and care partner.

The third and final component comprises *Outcomes*, categorized for patient, care partner, and dyad. Traditionally, survival and disease-specific outcomes for the patient are measured pre- to post-LVAD; these are important and, per ISHLT guidelines [11], outcomes should also include patient quality of life and psychosocial outcomes. Less understood are the care partner outcomes, for whom the intensity of the caring role may impact psychosocial factors, quality of life, health status, and feelings of meaningful contribution and partnership as well as the development of caregiving strain or burden. We deliberately included health status as an outcome in this model for care partners as caregiving burden can increase the risk of the care partners developing cardiovascular and metabolic disease [44, 45]. Unfortunately, the health of care partners is frequently overlooked. Finally, the dyad itself is impacted as an outcome (dyad as unit of analysis), as is recognized more among other populations living with chronic conditions (e.g., cancer, dementia). The science is not yet clear about the best identification of dyadic outcomes. To measure the impact of future interventions to improve outcomes for care partners, we proposed *mutuality* as a dyadic outcome, defined as “the positive quality of the relationship between the caregiver and receiver” [46] (p. 376). The interrelatedness of patient and care partner outcomes are represented by bidirectional arrows.

### Approaches to Dyadic Analysis

Collecting and reporting data from a patient and care partner does not make a study dyadic [47]. To move from conceptual frameworks to dyadic science, scientists must carefully consider the relationship between study variables for the patient, care partner, and dyad in study conception, measurement, outcomes, data analysis, and data interpretation. In study conceptualization there is a tension between patient, care partner, and dyadic outcomes and there should be careful consideration for who we are collecting data from (best practice is avoiding proxy measures; the patient and care partner often disagree) as well as what data is being collected with consideration of parallel measures when appropriate, with inclusion of health outcomes for the care partner. Bidwell et al. provided a comprehensive state of the science review of dyadic models applied among heart failure dyads [16]. Because care partner and patient relationships are interconnected, advanced statistical approaches are needed. Conventionally, the unit analysis for the patient-care partner dyad is a singular unit [48]. However, a pragmatic argument could be made that clinicians unfamiliar with advanced statistical approaches may benefit from having interpretable scores available for the individual patient and care partner as well as for the dyad as a unit. Lyons and Lee provided clarification of how multilevel dyadic models (matched pairs vs. incongruence model) can facilitate understanding of the dyadic experience of illness [48]. Future research may evaluate recommendations for clustering methods to find patterns in responses, and empirical Bayes estimates to examine congruence in outcomes to understand processes. A better understanding of the best approaches to dyadic analysis strengthens further understanding of congruence and patterns in a dyad.

## Implications for Research

### Conceptual Frameworks to Guide Research

In our proposed working framework, there are several areas that have yet to be elucidated, and we did not attempt to enter all self-management or LVAD dyadic management process variables in this framework, nor attempt to measure all in one study. Researchers should thoughtfully consider further adaptation of this and other frameworks to identify the processes by which LVAD patients, care partners, and dyads translate LVAD knowledge and skills into behaviors, as these processes may be amenable to intervention. While LVAD dyadic management is currently under-explored, work by Lorig and Holman [5] and Van de Veld et al. [49] in family management suggest *shared support*; *mutual support, patient-care partner-provider relationship; joint problem solving and decision making; joint action planning and evaluating outcomes; using available resources; and emotional management* are components. Research is also needed to characterize the multilevel barriers and facilitators of LVAD management for patients, care partners, and dyads.

### Sampling

Research is needed that includes LVAD patient-care partner dyads [50] and description of samples should include whether patients and care partners were a matched, dyadic sample vs. unmatched cohort sample. Further, reports need more transparency in describing whether patient and care partner were interviewed together or separately (which can affect disclosure); who said what quote; and who endorsed what theme. While challenging, evaluating the patient-care partner dyad at baseline (pre-LVAD implant) and post-LVAD allows for more accurate understanding of the dyadic self-management dynamic processes. For precision health, an accurate assessment is needed within the dyad to identify *who* is doing *what* aspects of self-management. LVAD management can put strain on a dyad as they negotiate new roles and responsibilities. Relationship quality needs further investigation regarding its association with care partner strain and patient quality of life; further investigation can demonstrate feasibility for interventions to improve relationship quality for these dyads (pre-, intra-, or postoperatively).

Variability in practice, payment, and precision healthcare by country and geographic locations exists, so researchers must clearly describe the settings. For example, after LVAD implant, in Australia, patients typically have longer inpatient length of stay compared to the U.S. to facilitate self-management needed prior to returning home, often to a remote area; and in Germany, patients routinely transition from hospital to a rehabilitation center [51]. Studies are needed to examine the impact of rehabilitation stays and tailored exercise interventions on care partners as well as patients.

### Interventions and Outcomes

The needs of LVAD patients and their care partners may continuously evolve over the course of their care, and this should be considered when developing and testing dyadic management interventions and selecting relevant outcomes. For example, in the postoperative days and weeks following implant, LVAD patients may lack physical or cognitive readiness to learn and perform skills, yet cognition and adherence predict quality of life [52]. Care partners are often responsible for a significant portion of LVAD management in the early days post-implant, with the assumption that LVAD patients gradually increase self-management skills. However, we have little data on when and how this process occurs and whether it occurs in all dyads. For example, patients with inadequate self-management skills pre-implant or patients who experience a perioperative stroke will have a very different post-operative trajectory, with different intervention and support needs.

In available studies, most LVAD patients were interested in gaining independence and acknowledge that they need ongoing support and help with LVAD management [23, 24, 29]. Simulation based LVAD self-management training improves LVAD self-management skills and knowledge [25]; however, it is unknown if this training changes LVAD dyadic management behaviors or increases independent LVAD self-management. Additionally, the skills gained during simulation training decline over time [53], warranting consideration for how intervention effects might be maintained. A recent multi-site study testing a 3-month self-management curriculum for LVAD patients improved patient depression but there was no statistically significant improvement in self-management [51]. Importantly, care partners had limited ability to participate in the curriculum due to the global pandemic and care partner outcomes were not assessed, highlighting an important area for future exploration [54]. Finally, it is important to note that many LVAD centers in the U.S. teach patients *not* to perform their own driveline site care due to assumptions that they will not be able to maintain aseptic technique [25]; yet many patients struggle to overcome limitations to independence while living with an LVAD [6].

Future dyadic management interventions should consider exploring dyadic awareness of dyadic management styles (congruency between care partners and patients, or lack thereof) as well as the degree to which self-efficacy can enhance dyadic communication. For example, the LVAD patient may work for or communicate to the care partner a need for more independence. Similarly, the care partner may set more limits on what they feel is expected of them within the dyad, allowing for more awareness of self as a person and not simply “vanishing” into the dyad. Interventional studies with heart transplant paired peers [55], heart failure dyads [56–59] and other dyads with chronic conditions [60, 61] may serve as stimuli for similar studies among LVAD dyads.

## Implications for Practice

### Pre-Implant Shared Decision-Making and Preparation

Infrastructure must support the dyad, not just the patient. Starting pre-implant, by providing more structured shared decision-making materials such as decision aids [62] in addition to traditional educational materials, clinicians may help dyads better align choice of therapy with preferences to help in their choice of whether to proceed with LVAD [11].

Because the care partner is not typically under the professional care of the LVAD clinician, the LVAD clinician may consider the care partner as “healthy” (or healthier, at least, at the time of implant) and underrecognize the subsequent impact of the LVAD implantation on the care partner. Despite guidelines calling for routine psychological assessment for care partners pre- *and* post-implant [11, 63, 64], the degree to which centers are implementing this is not well understood [65]. Using carefully selected, brief instruments in routine clinical care may promote an awareness of care partner status (e.g., anxiety, self-efficacy, health-related quality of life, or caregiver burden) so patients and care partners can recognize when they need more support. Moreover, routine physical assessment (e.g., blood pressure, cholesterol, sleep health) of the care partner is also needed because they are at increased risk of developing cardiovascular disease [44].

### Psychosocial, Educational Support, and Design Innovations

Connections with support and resources (e.g., professionally or peer led support groups, therapists) are critical, as well as identification of more than one support person. Unfortunately, currently home care nurses have often not appeared prepared to help with challenges, as reflected in a national survey [27]; strategies to prepare home care and rehabilitation healthcare staff are needed.

Multi-pronged approaches are needed, including advancing human-centered design to make self-management simpler (controller connections, apps and telehealth), more transparent expectations and precision in ongoing assessment for quality of dyadic management, and consideration for whether further resources are needed for the dyad.

### Dyadic Management Encompassing More than Device Management

Finally, beyond teaching skills to manage the LVAD device itself, clinician assessment and support of dyadic management needs to consider the ripple effects of an LVAD on multiple aspects of a dyad’s life (e.g., joint action planning and evaluating outcomes; emotional management; negotiating new roles, including the care partner role). Thus, collaboration of an interprofessional care team must be maximized. Further implications for practice are provided (Table 3), including recognizing who is at risk for suboptimal outcomes due to limited resources. LVAD self-management education must include coaching for patients and care partners to recognize and navigate role changes in the relationship (dependence/independence).

**Table 3:** Suggestions for research and practice for dyadic LVAD management

**Research:**
• Refine, apply, and test conceptual frameworks to guide research
• Characterize the multilevel barriers and facilitators of dyadic LVAD management
• Report on patient, care partner, and dyadic outcomes in LVAD research
• Clearly describe sampling (dyadic sample of matched pairs vs. unmatched sample)
• Describe whether patient and care partner were interviewed together or separately; who said what quote; and who endorsed what theme
• Evaluate the patient-care partner dyad at baseline (pre-LVAD implant) and post-LVAD to further describe the dyadic management trajectory
• Within the dyad, identify *who* is doing *what* aspects of self-management?
• Describe self-management processes (e.g., self-management tasks and skills) beyond managing the *device*
• Test how dyadic LVAD management impacts outcomes for patients, care partners, and the dyad
• Test dyadic LVAD management interventions
**Practice:**
• Connect care partners with support and resources
• Facilitate LVAD knowledge & skills among select home-care nurses and respite staff
• Use carefully selected, brief instruments in routine clinical care in real-time with the patient and care partner to promote:
○ Patient and care partner awareness of own outcomes (i.e., anxiety, depression, health-related quality of life, or caregiver burden)
○ Shared decision-making
• Administer parallel instruments at similar timepoints for patients and care partners to allow for comparisons along the LVAD trajectory
• When teaching dyadic LVAD management, include the following for the dyad:
○ Communication (e.g., about expectations, appraisal)
○ Dyadic LVAD management behaviors
○ Shared responsibility
○ Mutual support (reciprocity)
○ Patient-care partner-provider relationship
○ Use of available resources
○ Joint problem solving and decision making
○ Joint action planning and evaluating outcomes
○ Emotional management
○ Negotiating new roles (e.g., for patient: employment changes; for care partner: care partner role)

## Conclusion

In comparison to the heart failure population, we know very little about dyadic management among LVAD patients and care partners. A handful of primarily qualitative studies describe self-management tasks and skills but actual processes of dyadic management are less often studied, leaving a gap in understanding a) *who* is doing *what* at *which* timepoint, b) the respective outcomes for the patient, care partner, and the dyad, and c) which targeted interventions will best support outcomes for the patient, care partner, and the dyad. Consideration of the context is needed as dyads self-manage together with unique facilitators and barriers. Future studies should include both the care partner and LVAD patient, and should include thoughtfully selected frameworks to guide more inclusive future research in dyadic management among care partners and LVAD patients, with the ultimate goal of designing the *right intervention* for the *right person* at the *right time*.

## Key References

- Saeed D, Feldman D, Banayosy AE, Birks E, Blume E, Cowger J, et al. The 2023 International Society for Heart and Lung Transplantation Guidelines for Mechanical Circulatory Support: A 10- Year Update. *J Heart Lung Transplant*. 2023;42(7):e1–222.
  - ○ These international guidelines call for pre-LVAD implant and ongoing psychological assessment of patient and care partner.
- Schulman-Green D, Feder SL, David D, Rada L, Tesfai D, Grey M. A middle range theory of self- and family management of chronic illness. *Nurs Outlook*. 2023;71(3):101985.
  - ○ This paper offers a self- and family management framework that has been updated over several years, offering key insight into how management of a chronic condition may be shared.
- Lyons KS, Lee CS. The Theory of Dyadic Illness Management. *J Fam Nurs*. 2018;24(1):8–28.
  - ○ This paper offers an important dyadic theory, with promise for application and testing among LVAD dyads and subsequent potential to identify interventions and outcomes that are meaningful to patients, care partners, and clinicians.

## Supplementary Information

Below is the link to the electronic supplementary material.

Supplementary file1 (DOCX 20 KB)

## Data Availability

No datasets were generated or analysed during the current study.
