# Supplementary material for: Self and Dyadic Management of Patients and Care Partners Living with Durable Ventricular Assist Device: Emerging Science, Studies, and Frameworks to Advance Precision Health
Source: Curr Heart Fail Rep. 2025 Dec 20;23(1):2. doi: 10.1007/s11897-025-00737-6 (PMC12718229; doi:10.1007/s11897-025-00737-6)
Supplement: Supplementary file 1 — Supplementary file1 (DOCX 20 KB) [file 11897_2025_737_MOESM1_ESM.docx]

**Supplemental Tables**

For this purposive review, we sought to identify studies in which self-management outcomes were reported for both *person with an LVAD* and *caregiver of person with an LVAD.* We conducted online searches (with two different approaches). Studies were included regardless of whether the outcomes were analyzed by dyad or were unmatched patient/caregiver cohorts. Searches were limited to adults, English language, and within the past 10 years.

Studies were excluded if they only included patients OR only included caregivers. Studies analyzing posts on an LVAD social media site (myLVAD.com) were excluded because demographic data was not available to verify respondent’s role (e.g., who were patients vs. caregivers, vs. other) and count. Further exclusion criteria included reports that were abstract-only, and non-research clinical papers or opinion papers.

**Supplemental Table 1**

*Search Strategy 1*

| Ovid Search Self-care, self-mgt, caregivers, heart assist device.  OVID search  Ovid MEDLINE(R) ALL <1946 to February 05, 2025>  1 exp Self Care/ or exp Self-Management/ 71029  2 exp Caregivers/ 56204  3 exp Heart-Assist Devices/ 19065  4 exp Heart Transplantation/ 40990  5 1 and 2 and 3 12  A search was conducted combining the concepts of LVAD AND self care AND caregiver dyads using a combination of keyword and subject heading approaches (figure 1). Searches were updated the first week in April 2025 in the Medline, Embase, Cochrane Central Register of Controlled Trials, and Cochrane Database of Systematic Reviews databases via Ovid. Searches were limited to English language, adults, and publication date of 2015-2025.  Studies were included when self-management outcomes were reported for both *person with an LVAD* and *caregiver of person with an LVAD*, regardless of whether the outcomes were analyzed by dyad or were unmatched patient/caregiver cohorts. Studies were excluded if they only included patients OR only included caregivers. Studies analyzing posts on an LVAD social media site (myLVAD.com) were excluded because demographic data was not available to verify respondent's role (e.g, who were patients vs. caregivers, vs. other) and count. Further exclusion criteria included reports that were abstract-only, and non-research clinical papers or opinion papers. |
| --- |

**Table 1.1** Examples of Concepts and Search Terms

| Concept | Search terms |
| --- | --- |
| LVAD | "heart assist" or "left ventricular assist device*" or LVAD or LVADs |
| AND | |
| Self-management | caregiver outcome or self care or self management or self-reported outcome  "caregiver outcome*" or "self care*" or "self management" or "self-reported outcome*" |
| AND | |
| Caregiver dyad | "care giver*" or "care partner*" or caregiver* or dyad or dyads or families or family* |

**Supplemental Table 2**

*Search Strategy 2*

| Ovid  Database(s): EBM Reviews - Cochrane Central Register of Controlled Trials March 2025, EBM Reviews - Cochrane Database of Systematic Reviews 2005 to April 2, 2025, Embase 1974 to 2025 April 08, Ovid MEDLINE(R) and Epub Ahead of Print, In-Process, In-Data-Review & Other Non-Indexed Citations, Daily and Versions 1946 to April 08, 2025  Search Strategy:   \| **#** \| **Searches** \| **Results** \| \| --- \| --- \| --- \| \| 1 \| exp left ventricular assist device/ \| 26348 \| \| 2 \| ("heart assist" or "left ventricular assist device*" or LVAD or LVADs).ti,ab. \| 38014 \| \| 3 \| 1 or 2 \| 46095 \| \| 4 \| exp caregiver/ \| 195572 \| \| 5 \| ("care giver*" or "care partner*" or caregiver* or dyad or dyads or families or family*).ti,ab. \| 3067694 \| \| 6 \| 4 or 5 \| 3104468 \| \| 7 \| 3 and 6 \| 1054 \| \| 8 \| exp Self-Management/ \| 121830 \| \| 9 \| exp Self Care/ \| 187229 \| \| 10 \| ("caregiver outcome*" or "self care*" or "self management" or "self-reported outcome*").ti,ab. \| 155405 \| \| 11 \| 8 or 9 or 10 \| 266615 \| \| 12 \| 7 and 11 \| 84 \| \| 13 \| limit 12 to (english language and yr="2015 -Current") [Limit not valid in CDSR; records were retained] \| 72 \| \| 14 \| (newborn* or neonat* or infant* or toddler* or child* or adolescent* or paediatric* or pediatric* or girl or girls or boy or boys or teen or teens or teenager* or preschooler* or "pre-schooler*" or preteen or preteens or "pre-teen" or "pre-teens" or youth or youths).ti,ab,hw,kf. \| 10856355 \| \| 15 \| 13 not 14 \| 71 \| \| 16 \| limit 15 to (letter or conference abstract or editorial or erratum or note or addresses or autobiography or bibliography or biography or blogs or comment or dictionary or directory or interactive tutorial or interview or lectures or legal cases or legislation or news or newspaper article or overall or patient education handout or periodical index or portraits or published erratum or video-audio media or webcasts or conference review or clinical trial protocol or trial registry record) [Limit not valid in CCTR,CDSR,Embase,Ovid MEDLINE(R); records were retained] \| 31 \| \| 17 \| 15 not 16 \| 40 \| \| 18 \| remove duplicates from 17 \| 26 \| \| 19 \| from 18 keep 1-7, 9-16, 18, 20-25 \| 22 \| |
| --- | --- | --- | --- | --- | --- | --- | --- | --- | --- | --- | --- | --- | --- | --- | --- | --- | --- | --- | --- | --- | --- | --- | --- | --- | --- | --- | --- | --- | --- | --- | --- | --- | --- | --- | --- | --- | --- | --- | --- | --- | --- | --- | --- | --- | --- | --- | --- | --- | --- | --- | --- | --- | --- | --- | --- | --- | --- | --- | --- | --- |

Duplicate studies were excluded. Studies with a primary aim to evaluate an online app were further excluded. Studies were limited to past 10 years.
